# Anticancer activity of asiatic acid in cisplatin-resistant human neuroblastoma SH-SY5Y and its unaffected effect on human neural stem cells

**DOI:** 10.1371/journal.pone.0358134

**Published:** 2026-09-15

**Authors:** Apisada Jiso, Laphatrada Yurasakpong, Amarin Thongsuk, Tarinee Chodchavanchai, Nisarat Ruangsawasdi, Permphan Dharmasaroja, Anupong Thongklam Songsaad, Taweesak Tangrodchanapong

**Affiliations:** 1 Department of Pharmaceutical Sciences, Faculty of Pharmacy, Chiang Mai University, Chiang Mai, Thailand; 2 Chakri Naruebodindra Medical Institute, Faculty of Medicine Ramathibodi Hospital, Mahidol University, Samut Prakan, Thailand; 3 Department of Anatomy, Faculty of Science, Mahidol University, Bangkok, Thailand; 4 Department of Pharmacology, Faculty of Dentistry, Mahidol University, Bangkok, Thailand; 5 Department of Anatomy, Faculty of Dentistry, Mahidol University, Bangkok, Thailand; 6 School of Radiological Technology, Faculty of Health Science Technology, Chulabhorn Royal Academy, Bangkok, Thailand; Weill Cornell Medicine, UNITED STATES OF AMERICA

## Abstract

**Objective:**

To investigate the anticancer effects of asiatic acid (AA) and asiaticoside (AS) in cisplatin (Cis)-resistant neuroblastoma SH-SY5Y and neural stem cells derived from human stem cells from apical papilla (NSCs-hSCAPs).

**Methods:**

An MTT cell viability assay was performed to determine Cis toxicity and assess the cytotoxic effect of AA or AS on Cis-treated SH-SY5Y and the effect on NSCs-hSCAPs. Subsequently, the combination index (CI) was calculated to observe occurring interactions between the compound and Cis. For mechanistic exploration, flow cytometry using annexin V and PI staining and a caspase-3 activity assay were done to quantify apoptotic cell death. qRT-PCR was used to measure apoptotic and antioxidant mRNA levels. Additionally, the protein levels were analyzed by using the Western blot technique. Finally, to illustrate the putative interactions between the compound and its target, molecular docking was trialled.

**Results:**

MTT results revealed that AS did not affect both cells. However, 20 µM AA in combination with 2.5 µM Cis could significantly reduce SH-SY5Y cell viability with a CI value of 0.41, indicating their synergistic effect. Moreover, the effective concentration of AA did not affect Cis-treated NSCs-hSCAPs, suggesting its low toxicity. Considering SH-SY5Y, AA alone or its combination dramatically increased % apoptotic cells, which showed a minimal increment in Cis alone. Importantly, the combination treatment significantly increased caspase-3 activity. Mechanistically, *BAX* levels were elevated, whereas *BCL2, SOD-1*, and *HO-1* levels were downregulated by AA alone or in combination. These results were also consistent with Western blot analysis. Interestingly, an inhibition of GSK3β was observed in the combination treatment, suggesting that these outcomes may be related.The molecular docking illustrated that AA interacted with GSK3β and BCL-2 through multiple residues, indicating their potential binding interactions.

**Conclusion:**

AA and Cis treatment suppressed SH-SY5Y cell viability by enhancing apoptosis, reducing antioxidant gene expression, and potentially inhibiting GSK3β.

## Introduction

Brain metastases arising from cancers originating from neural crest progenitor or neural crest cells, such as neuroblastoma, melanoma, and malignant peripheral nerve sheath tumors (MPNSTs), showed a low median survival rate with poor prognosis [1–3]. The patients who have these malignancies express symptoms involving neurological and cognitive deficits. The ultimate goal for disease treatment is to palliate the symptoms and minimize their negative consequences [4]. Chemotherapy, including cisplatin (Cis) treatment, one of the therapeutic approaches, is utilized for brain metastasis. However, chemoresistance and chemobrain, a cognitive impairment that is caused by suppressing neuronal precursor and neural stem cell proliferation, have been described [4,5]. The mechanism by which brain metastasis resists Cis or other chemotherapeutic agents is mediated through subtherapeutic doses in the peripheral of a metastatic lesion, activation of cell adhesion receptors, angiogenesis, and a subpopulation of cells [6]. According to the literature, these biological processes may involve glycogen synthase kinase 3 beta (GSK3β), a serine/threonine kinase. GSK3 and GSK3β are highly expressed in neuroblastoma [7] and melanoma [8]. In these diseases, GSK3β has been associated with cancer cell proliferation, survival, migration, and invasion, which are prevented by its inhibition. Moreover, increased expression of GSK3β was observed in Cis-resistant ovarian cancer cells [9]. Additionally, GSK3β was found to activate Nuclear Factor kappa B (NF-κB) transcription factor in promoting the expression of antiapoptotic (B-cell lymphoma 2; BCL-2), and antioxidant proteins including superoxide dismutase (SOD), and heme oxygenase-1 (HO-1) that are needed for temoporfin resistance in head and neck squamous cell carcinoma (HNSCC) [10]. Taken together, GSK3β expression may be considered a crucial molecule in promoting tumorigenesis, metastasis, and chemoresistance of cancers originating from neural crest progenitor or neural crest cells that can spread to the brain as well as other tumors. Thus, its inhibition may be a potential therapeutic modality, especially in the case of brain metastasis with chemoresistance.

Asiatic acid (AA) and asiaticoside (AS), bioactive compounds from *Centella asiatica* (L.) Urban, exerted anti-cancer activity in many human malignancies [11,12]. In a previous report, AA at 75 µM demonstrated a dramatic decrease in cell viability of Cis-resistant nasopharyngeal NPC-039 and NPC-BM cell lines through activation of the intrinsic and extrinsic apoptotic pathway [12]. Furthermore, AA (10, 20, and 40 µM) could suppress transforming growth factor beta 1 (TGF‑β1) ‑induced epithelial-to-mesenchymal transition (EMT) in lung cancer A549 cells through increased expression of E‑cadherin. Moreover, the compound also reduced expression of GSK3β, a regulator of EMT [13]. Concerning AS, it has been shown to have anti-tumor effects in a variety of cancers, including melanoma [11,14]. Additionally, AS (1–10 µM) was able to inhibit cell proliferation, promote apoptosis, and cause G1 cell cycle arrest in chemotherapy-resistant hepatocellular carcinoma (HCC) cell lines QGY-7703 and Bel-7402 [11]. As evidenced previously, it is possible that AA and AS are highlighted candidates in developing as promising natural compounds to suppress cancer growth, metastasis, and chemoresistance mediated by GSK3β in brain metastases. Interestingly, both AA and AS highly passed through the blood-brain barrier (BBB) with high permeability of 70.61 ± 6.60 and 50.94 ± 10.91 x 10^−6^ cm/s, respectively [15]. Thus, they might be increased in the peripheral zones of brain metastatic lesion that is a leading cause of chemoresistance.

From these potentials, the present work aimed to investigate the anticancer effects of AA and AS in Cis-resistant neuroblastoma SH-SY5Y, a cancerous model expressing neuronal markers [16] that is acceptable for studying cell viability, apoptotic pathway, and metastatic properties [17]. In addition, to evaluate the potential neurotoxicity of the tested compounds toward non-malignant human neural cells, previously established human neural stem cell-like cells derived from stem cells from apical papilla (NSCs-hSCAPs) were cultured as a biologically relevant non-malignant neural cell model [18,19].

## Materials and methods

### Materials

**Fig 1:**
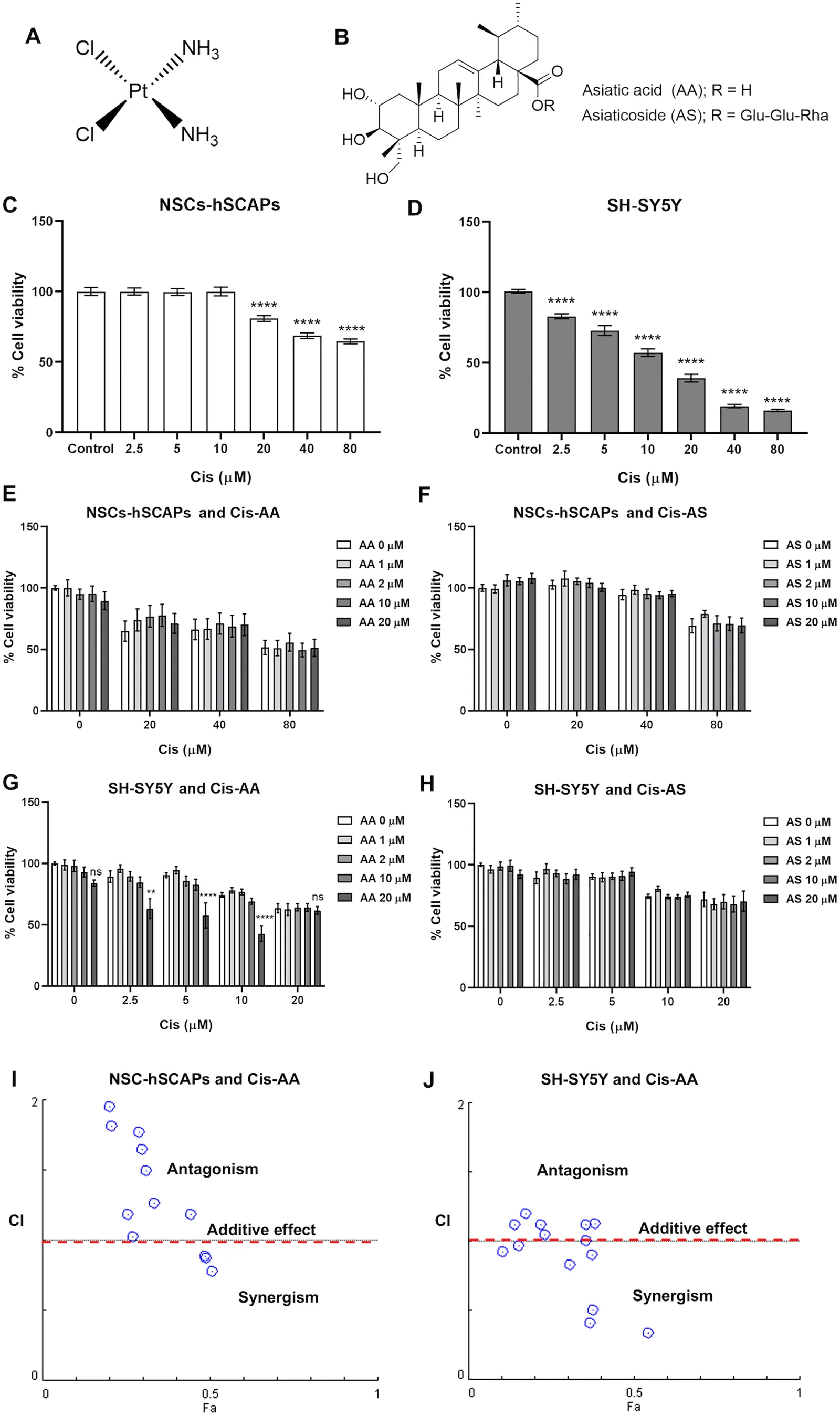
Effects of Cis and its combination with AA or AS on NSCs-hSCAPs and SH-SY5Y viability. **(A)** Chemical structure of Cis. **(B)** Chemical structures of AA and AS. **(C, D)** Cell viability (%) following 24-hour treatment with Cis (0–80 μM) in NSCs-hSCAPs and SH-SY5Y cells, respectively. **(E, F)** Cell viability (%) of NSCs-hSCAPs treated with Cis (0–80 μM) in combination with AA or AS (0–20 μM), respectively. **(G, H)** Cell viability (%) of SH-SY5Y cells treated with Cis (0–20 μM) in combination with AA or AS (0–20 μM), respectively. **(I, J)** The CI analysis for Cis-AA treatment in NSCs-hSCAPs and SH-SY5Y cells, respectively. Data are presented as mean cell viability (%) ± SEM from three independent experiments (n = 9/condition). Statistical significance was determined by comparison to control groups: **** = *p* < 0.0001 compared to untreated control (Fig 1C and 1D); ** = *p* < 0.01 compared to control (2.5 μM Cis alone), ****p < 0.0001 vs. control (5 or 10 μM Cis alone) (Fig 1G).

Cisplatin (Cis, CRS, C2210000), asiatic acid (AA, purity 97%, 546712), and asiaticoside (AS, purity ≥ 98.5%, 43191, Supelco®) were purchased from Sigma-Aldrich, MI, USA. The structures of Cis, AA, and AS were illustrated in Fig 1A and 1B.

### Cultivation of NSCs-hSCAPs

Neural stem cells induced from human stem cells derived from apical papilla (NSCs-hSCAPs) were prepared and characterized in a previous study [18] as a biologically relevant non-malignant neural cell model. This research protocol and ethical considerations were approved by the ethics review committee for the Human Rights Related to Human Experimentation of the Faculty of Dentistry/Faculty of Pharmacy and Faculty of Medicine Ramathibodi Hospital, Mahidol University, Thailand (MU-MOU COE 2023/003.1101, Protocol No. MU-MOU 2022/DT152), with further use in this current experiment. The procedure was conducted following the Declaration of Helsinki. Written informed consent was obtained for the experiment with human subjects during the recruitment period (11 January 2023–11 January 2025).

### Cultivation of human neuroblastoma cells (SH-SY5Y)

SH-SY5Y human neuroblastoma cells (CRL2266, ATCC, VA, USA) were cultured in a proliferation medium consisting of Dulbecco’s Modified Eagle Medium: Nutrient Mixture F-12 (DMEM/F-12, 12500-039, Gibco, Life Technologies, NY, USA), 10% Fetal Bovine Serum (FBS, A5256701, Gibco, Life Technologies), and 1% Antibiotics-Antimycotics (15240-062, Gibco, Life Technologies) at 37°C, 5% CO_2_, and 95% humidity incubator. The enzymatic digestion with trypsin-EDTA (15400-054, Gibco, Life Technologies) was performed to expand the cells for further experiments.

### Cell viability (MTT) assay

Cell viability was assessed through the MTT assay (3-(4,5-dimethylthiazol-2-yl)-2,5-diphenyl tetrazolium bromide, M5655, Sigma-Aldrich). The test compounds, including Cis, AA, and AS, were first dissolved in dimethyl sulfoxide (DMSO, D2650, Sigma-Aldrich), resulting in a concentration of 100 mM. Then, it was progressively diluted in DMEM/F-12 to create the final testing concentrations.Both NSCs-hSCAPs and SH-SY5Y cells were plated at a density of 30,000 cells/well in 96-well plates and incubated for 24 hours. The cells were subsequently exposed to various concentrations of Cis (0, 2.5, 5, 10, 20, 40, and 80 µM) or AA or AS(0, 1, 2, 10, 20, 50, 100, 250, and 500 µM) for 24 hours. Untreated control cells received only culture medium, while vehicle control groups were given medium containing 0.5% DMSO. After the treatment, the cells were exposed to MTT solution at 0.5 mg/mL for 2 hours. The resulting formazan crystals were dissolved using DMSO, and optical density measurements were taken at 570 nm (measuring solubilized formazan) and 690 nm (background correction) with a microplate reader (BioTek Epoch, Agilent, CA, USA). Cell viability (%) was calculated relative to untreated controls using a standard formula below. The IC_50_ values (The half-maximal inhibitory concentration) were calculated from dose-response curves using nonlinear regression analysis in GraphPad Prism software (LLC, MA, USA).


$$\begin{array}{c} \text{Cell viability }(\%)\;=\;\frac{\text{mean OD of treated group }(\text{OD570}-\text{OD690})}{\text{mean OD of control group }(\text{OD570}-\text{OD690})}\;x\;\text{1}\text{0}\text{0} \end{array}$$

The next step was to investigate the synergistic effect between AA or AS and Cis. In this experiment, the cells were pre-treated with non-toxic concentrations of AA or AS for 24 hours. After removing these compounds, the treated cells were incubated with Cis for another 24 hours. In turn, cell viability was assessed using the MTT assay, and the results were evaluated based on Equation 1. The significant cell viability (%) results obtained from the combination of AA or AS and Cis were further selected for calculating the combination index (CI).

### Calculation of the combination index (CI) of Cis

The CI of treatment in the cells was calculated using CompuSyn software (Biosoft, Ferguson, MO, USA) [20]. The fraction affected (Fa) was first defined according to the equation below and used to generate dose-effect curves for Cis and AA or AS. This method enabled a quantitative analysis of drug interactions using CompuSyn software, where CI > 1, = 1, and < 1 indicate antagonistic, additive, and synergistic effects, respectively.


$$\begin{array}{c} Fa=\frac{\text{1}\text{0}\text{0}-\%Cell\;viability}{\text{1}\text{0}\text{0}} \end{array}$$

### Flow cytometry

To discriminate between apoptotic, necrotic, and viable cells, the samples were treated with AA and Cis and consequently subjected to the Annexin V-FITC Kit (130-092-052, Miltenyi Biotec, Gladbach, Germany). Briefly, cells were harvested at a density of 500,000 cells/group and stained with Annexin V (5 µL) and propidium iodide (PI) (2.5 µL) to detect phosphatidylserine exposure and membrane permeability, respectively, which are hallmarks of early and late apoptosis [21]. The protocol was validated by the setup of unstained, Annexin V-stained, PI-stained, and Annexin V + PI-stained. Then, SH-SY5Y cells were treated with or without AA for 24 hours, followed by incubation with or without Cis for an additional 24 hours, which was represented as 1) the untreated group, 2) the cells treated with Cis alone, 3) the cells treated with AA alone, and 4) the cells treated with AA in combination with Cis. Consequently, an investigation of cell death was performed with the BD FACS Canto^TM^ Flow cytometer (BD Biosciences, CA, USA), counted at 20,000 events, and analyzed by BD FACSDiva^TM^ software (BD Biosciences).

### Caspase-3 activity

To demonstrate the typical hallmark of apoptosis detection, caspase-3 activity was performed. The tested samples were subjected to the Caspase-3 Activity Assay Kit (5732S, Cell Signaling Technology, MA, USA) according to the manufacturer's instructions. Briefly, SH-SY5Y cells were treated under the same experimental conditions as described above. Following treatment, the caspase-3 fluorogenic substrate was added and incubated at 37°C in the dark. Fluorescence intensity was measured using a Synergy™ H1 Hybrid Multi-Mode Microplate Reader (BioTek) at an excitation wavelength of 380 nm and an emission wavelength of 420−460 nm. Caspase-3 activity was expressed as the fold increase in relative fluorescence intensity, which was calculated using the following equation:


$$\begin{array}{c} \text{Fold increase in relative fluorescence intensity}=\frac{\text{fluorescence intensity of experiment group}}{\text{fluorescence intensity of control group}} \end{array}$$

### Quantitative reverse transcription polymerase chain reaction (qRT-PCR)

To investigate the underlying mechanisms of AA and/ or Cis in reducing cell viability (%) and inducing apoptosis of SH-SY5Y cells, qRT-PCR was carried out. In this experiment, the cells were divided into 4 groups as follows: 1) the untreated group, 2) the cells treated with Cis alone, 3) the cells treated with AA alone, and 4) the cells treated with AA in combination with Cis. After the treatment for each condition, the cells in those groups were harvested for RNA extraction using FavorPrep™ Tissue Total RNA Purification Mini Kit (FATRK 001-1, Favorgen Biotech Corp., Pingtung, Taiwan) following the manufacturer’s protocol. The collected RNA levels were measured by NanoDrop™ 2000/2000c spectrophotometer (Thermo Scientific, MA, USA), and their purity was evaluated by UV absorbance (260/280 and 260/230 ratio). The RNA samples were converted to cDNA through the iScript™ Reverse Transcription Supermix (#1708841, Bio-Rad, CA, USA) reaction. Quantitative PCR was done in a 20 µL mixture solution of KAPA SYBR™ FAST qPCR Master Mix (2X) Kit (KK4600, Kapa Biosystems, Cape Town, South Africa), PCR-grade water, specific primers, and the cDNA. The primer sequences utilized in this technique are demonstrated in Table 1. PCR reactions were run in a CFX96 Touch Real-time PCR machine (CFX96 Touch, Bio-Rad) under the following conditions: the reaction was started with 1 cycle of 95°C for 3 minutes, followed by 40 cycles at 95°C for 3 seconds and 60°C for 30 seconds, and melting curve analysis according to the manufacturer’s procedure. The Cq values were calculated with equation 2^-ΔΔCq^ for evaluating fold change in the expression of each gene. The expression levels of mRNA were normalized using glyceraldehyde-3-phosphate dehydrogenase (*GAPDH*). Data were obtained from three independent experiments.

**Table 1:** Oligonucleotide primer sequences for genes used in this experiment.

Gene	Accession No.	Oligonucleotide primer sequences (5’◊3’)
*HO-1*	NM_002133.3	**Forward**: AAACTTCAGAGGGGGCGAAG
		**Reverse**: GCTGCCACATTAGGGTGTCT
*SOD-1*	NM_000454.5	**Forward**: GCTGGAAGTCGTTTGGCTTG
		**Reverse**: AGGGGCCTCAGACTACATCC
*BCL2*	NM_000633.3	**Forward**: AAAAATACAACATCACAGAGGAAGT
		**Reverse**: ACGAGGGGGTGTCTTCAATC
*BAX*	NM_138761.4	**Forward**: GGCCCTTTTGCTTCAGGGT
		**Reverse**: CACTCGCTCAGCTTCTTGGT
*GSK3β*	NM_002093.4	**Forward**: ATTTCACCTCAGGAGTGCGG
		**Reverse**: AAGAGTGCAGGTGTGTCTCG
*GAPDH*	NM_001256799.3	**Forward**: GAAAGCCTGCCGGTGACTAA
		**Reverse**: GCATCACCCGGAGGAGAAAT

### Western blot

To quantify the expression levels of relevant proteins involved in apoptosis of the tested SH-SY5Y cells, the Western blot technique was implemented. Firstly, the treated cells were lysed in RIPA buffer mixed with Phenylmethanesulfonyl Fluoride (PMSF) (8553S, Cell Signaling Technology) for 5 minutes. The samples were then centrifuged at 18,000 g at 4°C for 10 minutes. Equal amounts of total protein (15 µg), as measured using a Pierce™ BCA Protein Assay Kit (23225, Thermo Scientific), were boiled before electrophoresis at 95°C for 10 minutes. After cooling with ice, the protein samples were loaded on polyacrylamide gels and run at 120 V in running buffer for 90 minutes. The gels were transferred to 0.45 µm nitrocellulose membranes (#1620115, Bio-Rad) using a transfer buffer with a constant amplitude at 220 mA for 120 minutes. Blots were blocked in 5% skim milk for 60 minutes. The membranes were then washed with Tris-Buffered Saline with Tween 20 (TBST). The interest proteins were detected by GSK3β (3D10) Mouse mAb (1:1,000) (9832S), Phospho-GSK3β (Ser9) (D2Y9Y) Mouse mAb (1:1,000) (14630S), BCL2 (124) Mouse mAb (1:1,000) (15071S), BAX (2D2) Mouse mAb (1:1,000) (89477S), and GAPDH (D4C6R) Mouse mAb (1:1,000) (97166S). Anti-mouse IgG, HRP-linked antibody (1:2,500) (7076S) was used as the secondary antibody. All specific antibodies used in this study were purchased from Cell Signaling Technology. Positive bands were developed with the enhanced chemiluminescence (ECL) method and captured by a chemiluminescent gel document (Amersham™ ImageQuant 800, Cytiva, MA, USA). The mean densities of protein signals were analyzed using the Image-J software (National Institutes of Health, MD, USA). The representative data were obtained from three independent experiments.

### Molecular docking

To explore the potential binding interaction between AA and GSK3β or BCL-2. The three-dimensional (3D) molecular structure of AA was obtained from PubChem (PubChem CID 119034). The crystal structures of GSK3β and BCL-2 were obtained from the Protein Data Bank (PDB) PDB ID 1I09 and PDB ID 4IEH, respectively. The molecular docking of AA to either GSK3β or BCL-2 was initially prepared using UCSF Chimera (The Resource for Biocomputing, Visualization, and Informatics (RBVI) at the University of California, San Francisco (UCSF), CA, USA). Subsequently, the interactions were evaluated using the BIOVIA Discovery Studio Visualizer (BIOVIA, Dassault Systèmes, BIOVIA Discovery Studio Visualizer, Version 20.1.0.192, SD: Dassault Systèmes, CA, USA). Rendered images of the protein-ligand interaction of the docked complexes were produced using PyMOL version 2.4.1 (Schrödinger, Inc., NY, USA). Root mean square deviation (RMSD) values and types of interactions were evaluated.

### Statistical analysis

The data were reported as the mean ± standard error of the mean (SEM) or standard deviation (SD) from three independent experiments. Statistical analysis was conducted using One-way ANOVA with Tukey’s multiple comparisons in GraphPad Prism version 10.5.0 (673) (GraphPad Software, LLC, MA, USA). A *p*-value of less than 0.05 was considered statistically significant.

## Results

### Effect of Cis on NSCs-hSCAPs and SH-SY5Y cell viability

Cell viability (%) was assessed following 24-hour exposure to Cis at doses ranging from 0 to 80 µM in both NSCs-hSCAPs and SH-SY5Y cells. NSCs-hSCAPs treated with lower concentrations of Cis (2.5–10 µM) showed no significant reduction in their cell viability (%). However, at higher concentrations (20–80 µM), cytotoxic effects became apparent (*p* < 0.0001), and the IC_50_ value of 120.5 µM was determined (Fig 1C).

Considering Cis toxicity in SH-SY5Y cells, a significant reduction of cell viability (%) was exhibited when the cells were tested with concentrations ranging from 2.5 to 80 µM (*p* < 0.0001) (Fig 1D). The IC_50_ value was 12.84 µM. Thus, the lower concentrations of Cis (2.5–20 µM) that could produce a significant decrease in SH-SY5Y cell viability were selected for further investigation. Additionally, compared with NSCs-hSCAPs, greater toxicity in SH-SY5Y was observed.

### Effect of AS or AA on Cis-treated NSCs-hSCAPs and SH-SY5Y

To determine the effects of AA or AS on NSCs-hSCAPs or SH-SY5Y cells treated with Cis, the cells were pretreated for 24 hours with AA or AS (1, 2, 10, or 20 µM) followed by Cis exposure for another 24 hours. In our previous study, nontoxic concentrations of either AS or AA were 1–20 µM for the cells [18]. Cis concentrations were adjusted based on cell line, including 20–80 µM for NSCs-hSCAPs and2.5–20 µM for SH-SY5Y cells. NSCs-hSCAPs showed no statistical differences in cell viability (%) when compared between the untreated group and the combination treatment group (Fig 1E and 1F). This indicated that nontoxic concentrations of AS or AA did not affect NSCs-hSCAPs.

In SH-SY5Y cells, AS at nontoxic concentrations had no effect on decreasing cell viability (%) (Fig 1H). However, a significant reduction of cell viability (%) was observed when the cells were treated with 20 µM AA and Cis in concentrations of 2.5, 5, and 10 µM, with *p*-values = 0.0011, <0.0001, and <0.0001, respectively (Fig 1G). Thus, it is plausible that AA might possess an anticancer effect in Cis-treated SH-SY5Y cells.

### Combination index of AA and Cis-treated NSCs-hSCAPs and SH-SY5Y

To assess the combinatorial effects of AA and Cis on NSCs-hSCAPs and SH-SY5Y cells, the CI analysis was conducted using CompuSyn software. In NSCs-hSCAPs, three synergistic interactions (CI < 1) were observed exclusively at high Cis concentrations (80 μM) when combined with 1, 10, and 20 μM AA (S1 Table and Fig 1I). Conversely, lower Cis concentrations produced antagonistic effects (CI > 1). It is indicated that Cis in a high concentration of 80 μM should not be used in combination with AA, whereas its lower concentrations showed an unaffected effect on AA-treated NSCs-hSCAPs.

SH-SY5Y cells demonstrated more favorable combination profiles (S2 Table and Fig 1J). One combination (2 μM AA and 10 μM Cis) exhibited additive effects (CI = 1). Seven combinations showed synergistic interactions (CI < 1) when 1–20 μM AA was combined with 2.5–20 μM Cis. The most potent synergistic effect was achieved with 20 μM AA and 10 μM Cis (CI = 0.33), while the remaining combinations displayed antagonistic interactions.

These CI analyses support the therapeutic potential of AA-Cis combinations in cancer treatment strategies. Based on optimal synergistic efficacy and a favorable safety profile, the 20 μM AA + 2.5 μM Cis combination (CI = 0.41) was selected for further mechanistic investigation.

### Discrimination of apoptosis by flow cytometry

**Fig 2:**
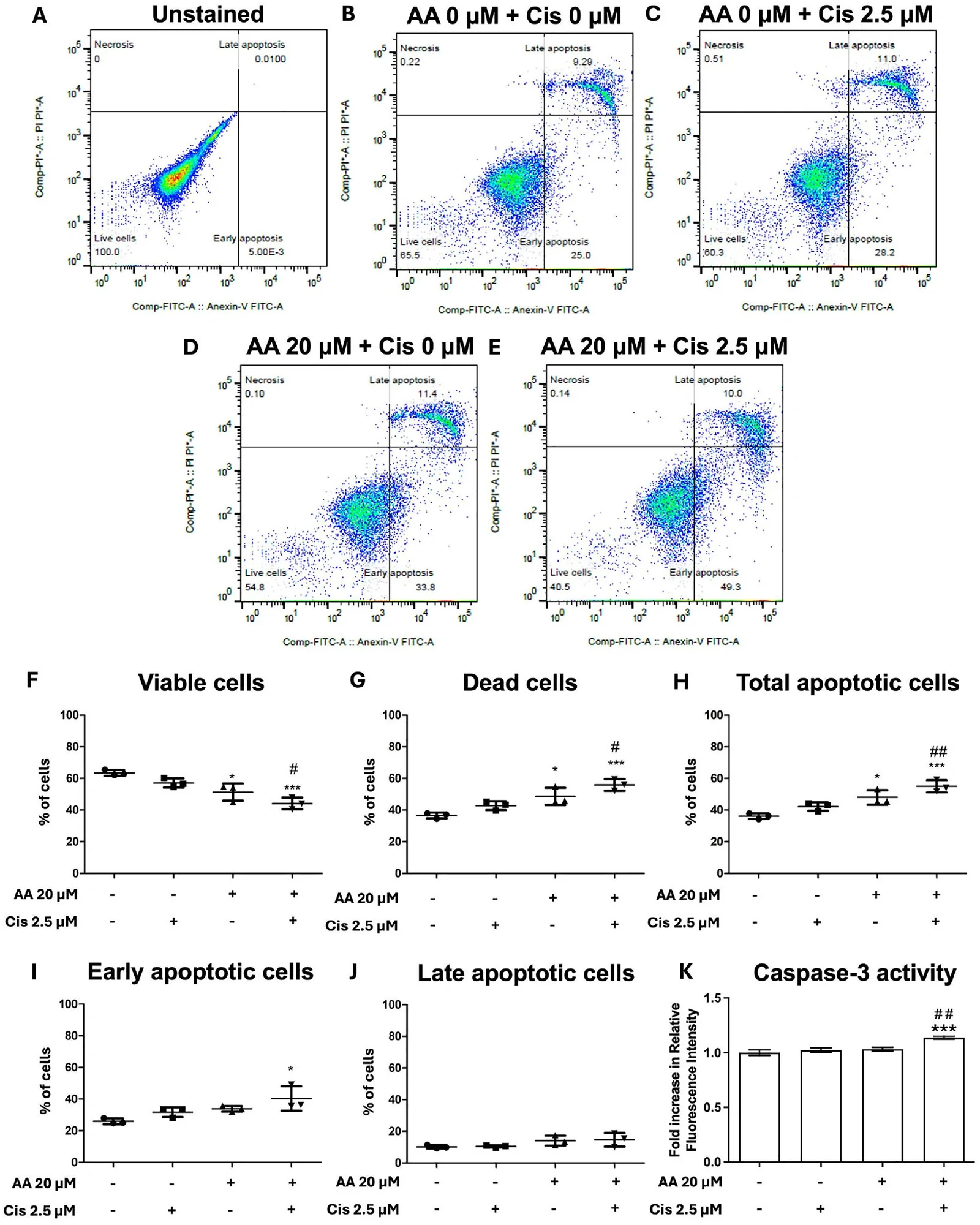
Discrimination of cell death and caspase-3 activity. The dot plot between Annexin-V and PI of various experimental groups. **(A)** unstained, **(B)** AA 0 µM + Cis 0 µM, **(C)** AA 0 µM + Cis 2.5 µM, **(D)** AA 20 µM + Cis 0 µM, **(E)** AA 20 µM + Cis 2.5 µM, **(F)** The (%) of viable cells. **(G)** The (%) of dead cells. **(H)** The (%) of total apoptotic cells. **(I)** The (%) of early apoptotic cells. **(J)** The (%) of late apoptotic cells. Results are represented as % of cells ± SD from three independent experiments. The number of samples is n = 9 for each condition. Error bars represent SD. * = *p* < 0.05, *** = *p* < 0.001 compared to the control group (AA 0 µM + Cis 0 µM), # = *p* < 0.05, ## = *p* < 0.01, compared to the Cis group (2.5 µM Cis alone). **(K)** The fold increase in relative fluorescence intensity in various experimental groups, including AA 0 µM + Cis 0 µM, AA 0 µM + Cis 2.5 µM, AA 20 µM + Cis 0 µM, and AA 20 µM + Cis 2.5 µM. Results are represented from three independent experiments. The number of samples is n = 9 for each condition. Error bars represent SEM. *** = *p* < 0.001 compared to the control group (AA 0 µM + Cis 0 µM), ## = *p* < 0.01, compared to the Cis group (2.5 µM Cis alone).

To evaluate the effects of AA and Cis on apoptosis, an Annexin V/PI assay was performed and analyzed by flow cytometry. As revealed by the dot plot graph (Fig 2A – 2E), in the untreated control group (AA 0 µM + Cis 0 µM), the majority of cells remained viable, indicating minimal baseline apoptosis when compared to the unstained group (Fig 2A). The viable cells were significantly decreased and reached the lowest in the combined group (AA 20 µM + Cis 2.5 µM) (*p* < 0.001), when compared to the untreated group and the Cis alone group (AA 0 µM + Cis 2.5 µM) (*p* < 0.05) (Fig 2F). Importantly, the dead cells, total apoptotic cells, and early apoptotic cells showed an increasing trend when compared to the control (Fig 2G–2I). Whereas the late apoptotic population was observed as less than 20% (Fig 2J). Treatment with 2.5 µM Cis alone led to a slight increase in dead cells, total apoptotic cells, early apoptotic cells, and late apoptotic cells, which may indicate Cis resistance of SH-SY5Y cells (Fig 2G–2I). Notably, the SH-SY5Y cells treated with 20 µM AA alone (AA 20 µM + Cis 0 µM) showed a significant increase in dead cells and total apoptotic cells from the control (*p* < 0.05), suggesting the potential of AA to promote cell death in SH-SY5Y cells (Fig 2G–2I). Interestingly, the combination of 20 µM AA and 2.5 µM Cis significantly represented the highest dead cells (*p* < 0.001), total apoptotic cells (*p* < 0.001), and early apoptotic cells (*p* < 0.05), indicating a synergistic effect that enhanced Cis-induced apoptosis (Fig 2G–2I).

### Caspase-3 activity

To further validate apoptosis induced by the combination treatment, caspase-3 activity was evaluated by measuring the relative fluorescence intensity, expressed as the fold increase over the untreated control. Treatment with Cis alone (2.5 µM) or AA alone (20 µM) did not significantly affect caspase-3 activity. Importantly, the combination treatment significantly increased caspase-3 activity compared with both the untreated control (*p* < 0.001) and the Cis-treated group (*p* < 0.01) (Fig 2K). These findings may support an orthogonal hallmark of apoptosis induced by the combination treatment.

### Effect of AA on antioxidant and apoptotic gene levels in Cis-treated SH-SY5Y

**Fig 3:**
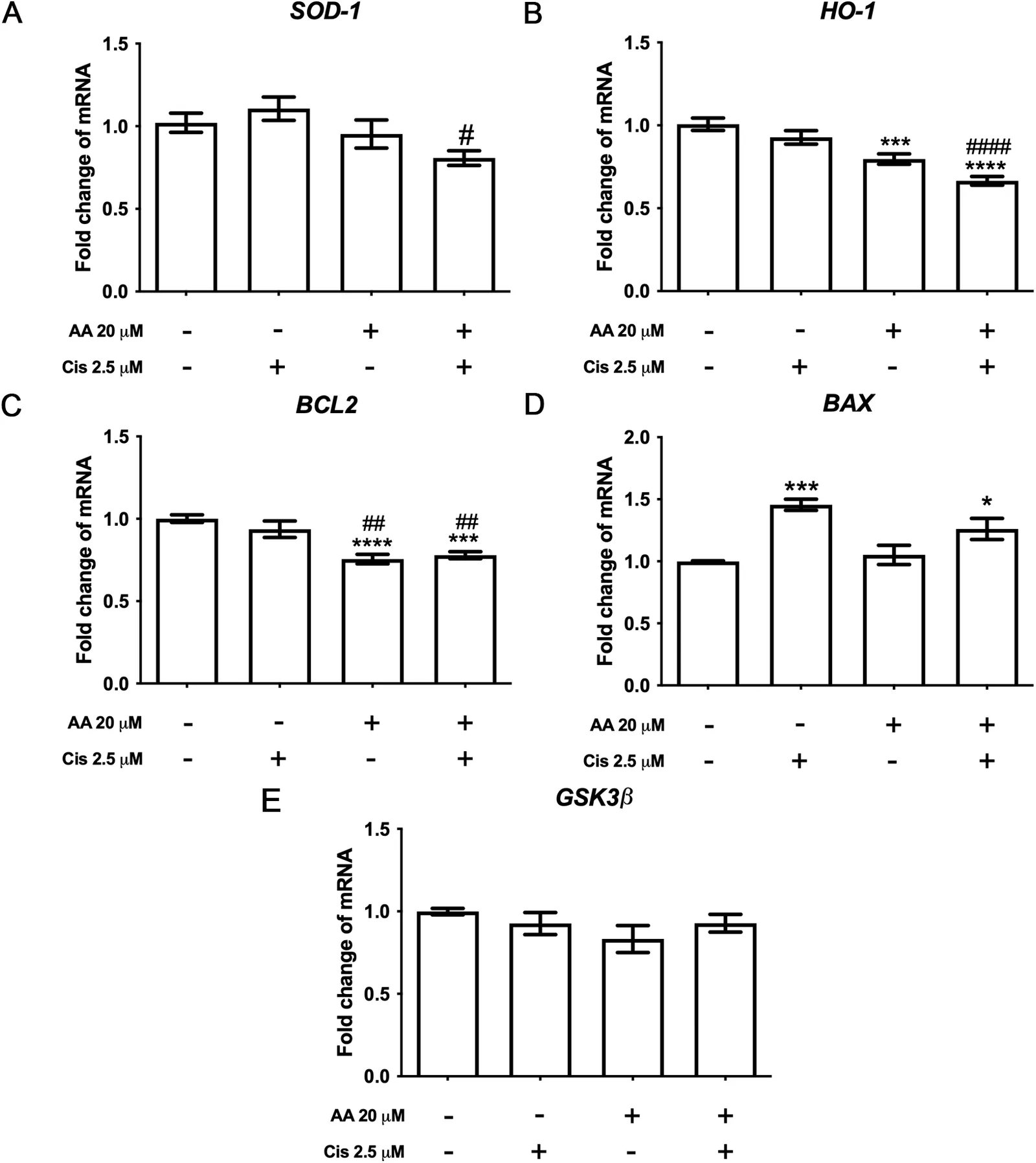
Effects of AA, Cis, or their combination on antioxidant and apoptotic gene expression in SH-SY5Y. mRNA levels of **(A)** *SOD-1*, **(B)** *HO-1*, **(C)** *BCL2*, **(D)** *BAX*, and **(E)** *GSK3β*. All mRNA expression levels in the cells were quantified by qRT-PCR at 24 hours after Cis treatment and normalized with the internal control *GAPDH*. Data are expressed as mean ± SEM from three independent experiments. The number of samples is n = 9 for each condition. ** = *p* < 0.01, *** = *p* < 0.001, and **** = *p* < 0.0001 vs. untreated cells. ^#^ = *p* < 0.05, ^##^ = *p* < 0.01, ^####^ = *p* < 0.0001 vs. Cis-treated cells.

To explore the possible mechanisms of AA and/or Cis in reducing cell viability and apoptosis in SH-SY5Y, the gene expression levels of *SOD-1, HO-1*, *BCL2*, and *BAX* were measured using qRT-PCR. The results showed that the expression levels of *SOD-1* mRNA were slightly increased in Cis-treated SH-SY5Y cells (Fig 3A). However, these were found to be decreased when the cells were administered 20 µM AA, and its combined treatment with 2.5 µM Cis. Interestingly, in a group of AA in combination with Cis, a significant downregulation of *SOD-1* was obtained when compared to Cis alone (*p* < 0.05). Concerning the *HO-1* mRNA expression level, Cis at a concentration of 2.5 µM did not reduce the expression of the gene compared to the untreated group. Nevertheless, AA alone and its combination with Cis could significantly downregulate such genes with *p* < 0.001 and *p* < 0.0001, respectively. Moreover, a dramatic reduction of *HO-1* mRNA levels was observed in the combination treatment compared to Cis alone (Fig 3B). From these findings, it is indicated that Cis alone could not minimize the levels of antioxidant *SOD-1* and *HO-1* genes in the tested SH-SY5Y, suggesting its resistance to the chemotherapy. On the other hand, a combination of AA and Cis demonstrated a strong effect in reducing the expression of those genes, particularly *HO-1.*

In addition to *SOD-1* and *HO-1*, the expression levels of *BCL2* and *BAX* were also affected after treatment to the cells. In comparison to the untreated group, *BCL2* mRNA levels were not changed in Cis-treated cells, while AA alone or its combination with Cis was able to produce a significant reduction with *p* < 0.0001 and *p* < 0.001, respectively (Fig 3C). Moreover, either the treatment with AA alone or the combined treatment was able to significantly decrease the *BCL-2* levels when compared to Cis alone (*p* < 0.01). Interestingly, the combined treatment could significantly upregulate the *BAX* mRNA expression levels, as shown in Fig 3D. It is likely that AA or its combination with Cis could promote SH-SY5Y apoptosis. In this present work, an upstream molecule of BCL-2, GSK3β, was also investigated at a genetic level. The results revealed that there was no statistically significant difference in the number of *GSK3β* genes compared between Cis-treated cells and untreated cells (Fig 3E). However, it was noticeable that the cells in a group of AA alone contained lower gene expression when compared to untreated cells and the cells incubated with Cis alone. This suggested that AA might affect *GSK3β* gene expression.

### Effect of AA on apoptotic protein and GSK3β levels in Cis-treated SH-SY5Y

**Fig 4:**
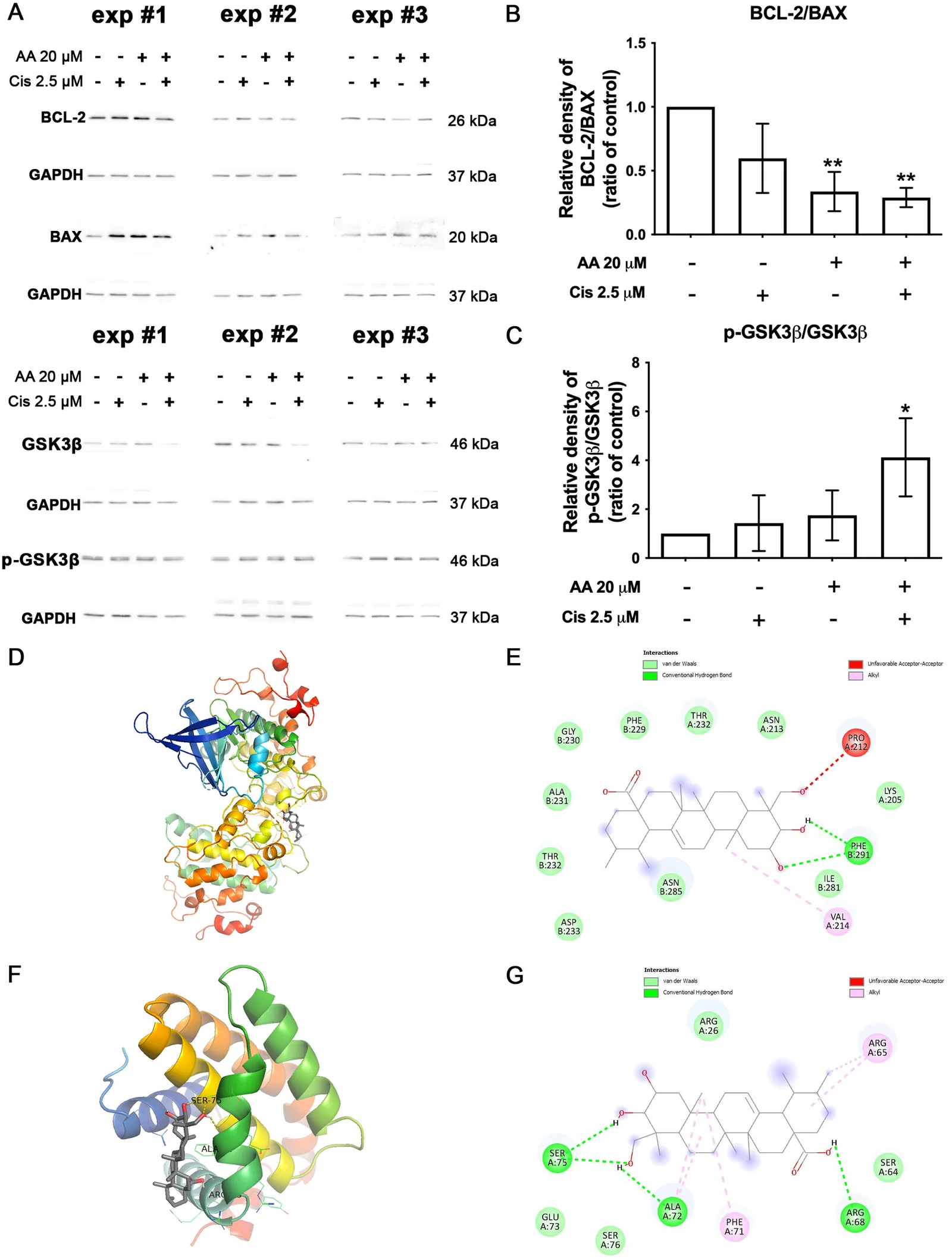
Effects of AA or its combination on apoptotic proteins and GSK3β and its potential interaction. Representative Western blot of BCL-2 (26 kDa), BAX (20 kDa), GSK3β (46 kDa), p-GSK3β (Ser9) (46 kDa), and GAPDH (37 kDa) in the tested cells (A). The levels of the BCL2/BAX and the p-GSK3β/GSK3β bands obtained from untreated and treated cells were quantified in (B) and (C), respectively. Data are shown as the mean ± SD of the band density from three independent experiments. * = *p* < 0.05, and ** = *p* < 0.01 vs. untreated cells. Molecular docking model in 3D of AA binding to GSK3β (D) or BCL-2 (F). 2D docking images illustrated different types of binding interaction between AA and GSK3β (E) or BCL-2 (G).

To quantify the expression levels of proteins involved in apoptosis, GSK3β, and p- GSK3β, in SH-SY5Y cells, the Western blot technique was implemented. The positive bands of BCL-2 (26 kDa), BAX (20 kDa), GSK3β (46 kDa), and p-GSK3β (46 kDa) obtained from the control, Cis alone, AA alone, or their combined treatment were illustrated in Fig 4A and quantified in Fig 4B, 4C. In Fig 4B, the ratio of BCL2/BAX did not show a significant decrease compared between untreated cells and Cis-treated cells. On the contrary, in comparison to untreated cells, statistical differences in such a ratio were observed when AA alone or its combined treatment was administered to the cells (*p* < 0.01). The ratio was the most reduced in the combination regimen. Moreover, the levels of BCL2/BAX tended to be lower in the group of AA alone and the combination treatment compared to the group of Cis alone. These indicated that AA might possess its effect to induce apoptosis in the cells treated with Cis.

In addition to BCL-2/BAX, the levels of GSK3β, an upstream molecule of BCL-2, and p-GSK3β (Ser 9), which is an inhibitory form of GSK3β, were also examined in SH-SY5Y cells. The p-GSK3β/GSK3β ratio was observed. In Fig 4C, Cis-treated cells showed only a minimal increase in p-GSK3β/GSK3β levels compared to untreated cells. However, this increment was higher in AA alone and reached statistical significance in the combination treatment (*p* < 0.05). These results suggest that the combination of AA and Cis may suppress the GSK3β activity.

### Potential chemical interactions of AA and its targets

To observe the *in silico* interactions between AA and GSK3β or BCL-2, the molecular docking was carried out. Fig 4D illustrates the putative interaction of AA with the crystal structure of GSK3β. It possibly interacted with the GSK3β molecule at residue phenylalanine 291 (PHE291) through two hydrogen bonds (Fig 4E). Furthermore, it formed Van der Waals forces with lysine 205 (LYS205), asparagine 213 (ASN213), threonine 232 (THR232), PHE229, glycine 230 (GLY230), alanine 231 (ALA231), THR232, aspartic acid 233 (ASP233), ASN285, and isoleucine 281 (ILE281). In addition, AA interacted with valine 214 (VAL214) by an alkyl bond. The binding energy was −6.2 kcal/mol. The RMSD value was 0.624 Å, suggesting an acceptable orientation of its binding.

In addition to GSK3β, AA could interact with BCL-2 at certain residues, including arginine 68 (ARG68), alanine 72 (ALA72), and serine 75 (SER75) by hydrogen bonds (Fig 4F and 4G). Additionally, it could bind to arginine 26 (ARG26), serine (SER64), glutamic acid 73 (GLU73), and SER76 via Van der Waals forces as well as PHE71 through an alkyl bond. The binding energy was −8.0 kcal/mol. The RMSD value was 0.061 Å, which is less than 2 Å from three trials; thus, such binding orientation was deemed acceptable and closely resembled the native binding mode.

## Discussion

This study examined the effects of AA or AS on NSCs-hSCAPs and SH-SY5Y treated with Cis. From our observation, Cis at 0–10 µM (IC_50_ > 100 µM) showed no cytotoxicity in NSCs-hSCAPs, but its higher concentrations at 20–80 µM could significantly reduce cell viability (%). In contrast to NSCs-hSCAPs, SH-SY5Y cells were more toxic to Cis, showing a significant reduction in cell viability (%) at 2.5–80 µM with an IC_50_ of 12.84 µM. Non-toxic concentrations of AA or AS showed no lethal effect in Cis-treated NSCs-hSCAPs. However, in SH-SY5Y cells, pre-treatment with 20 µM AA significantly increased Cis cytotoxicity at concentrations of 2.5, 5, and 10 µM. This indicated that AA selectively chemosensitizes cancer cells without affecting the non-malignant neural cell model.

To evaluate the potential neurotoxicity of the tested compounds, NSCs-hSCAPs were employed as a biologically relevant non-malignant human neural stem-like cell model. These cells have been previously established and extensively characterized, demonstrating the expression of canonical neural stem cell markers (Nestin and SOX2), self-renewal through 3D neurosphere formation, and the capacity to differentiate into neuronal-like cells following neurogenic induction. Furthermore, NSCs-hSCAPs have previously been applied to investigate neural differentiation, neuronal maturation, and the effects of therapeutic compounds on human neural stem-like cells [18,19,22]. Therefore, they provide a biologically relevant *in vitro* model for evaluating the safety profile of candidate therapeutic compounds toward non-malignant human neural stem-like cells.

To further evaluate the effectiveness of Cis and AA combination treatment, the CI values using CompuSyn software were calculated. The analysis identified seven instances in which Cis and AA displayed a synergistic effect. Moreover, their combination impacts were greater than the effect of either Cis or AA alone. Only one combination demonstrated additive effects, while the others exhibited antagonistic interactions. Based on the synergistic CI analysis, Cis 2.5 µM + AA 20 µM, which had the optimal CI index, was then selected for further mechanistic investigation.

Mechanistically, AA significantly induced apoptosis in SH-SY5Y cells and enhanced Cis-induced cell death when treated in combination. Flow cytometry analysis revealed that treatment of Cis alone (2.5 µM) induced only a modest apoptotic effect, suggesting a relative resistance of SH-SY5Y cells to Cis. Furthermore, the euroblastoma cells frequently develop intrinsic or acquired resistance to platinum-based chemotherapy, thereby limiting its clinical efficacy [23]. The apoptosis assay via Annexin V/PI staining further substantiates these results. A substantial increase in total apoptotic populations was observed in the combined treatment group (AA 20 µM + Cis 2.5 µM), indicating that AA may sensitize SH-SY5Y cells to Cis by promoting apoptosis. These results suggest a potential therapeutic benefit of combining AA with Cis in neuroblastoma treatment.

To further strengthen the evidence for apoptosis, a caspase-3 activity assay was performed as an orthogonal method to complement the Annexin V/PI flow cytometric analysis. Caspase-3 is a key executioner caspase activated downstream of mitochondrial outer membrane permeabilization and is tightly regulated by the balance between pro-apoptotic and anti-apoptotic BCL-2 family proteins [24]. Interestingly, although AA alone (20 µM) increased apoptotic cell death and modulated apoptosis-related molecular markers, including *BCL2* expression, the BCL-2/BAX protein ratio, and p-GSK3β/GSK3β, it did not produce a statistically significant increase in caspase-3 activity compared with the untreated control. Similarly, Cis alone (2.5 µM) did not significantly activate caspase-3 in Cis-resistant SH-SY5Y cells. Notably, the combination of AA and Cis significantly increased caspase-3 activity compared with either treatment alone, which was consistent with the greater apoptotic cell population detected by Annexin V/PI flow cytometry and the decreased BCL-2/BAX ratio observed by Western blot analysis. Collectively, these findings provide complementary evidence that AA enhances Cis-induced apoptosis and functions as a chemosensitizer in Cis-resistant SH-SY5Y cells. Nevertheless, this study did not include pharmacological inhibition of apoptosis, such as treatment with a pan-caspase inhibitor, which could provide additional mechanistic evidence to confirm whether the observed cell death and downstream molecular changes are caspase-dependent. Therefore, the absence of apoptosis inhibitor experiments warrants further investigation in future studies.

At the genetic and protein levels, SH-SY5Y cells treated with Cis alone had no effect in reducing the mRNA expression levels of antioxidant *SOD-1* and *HO-1,* as well as anti-apoptotic *BCL2*. Similarly, the Western blot analysis revealed that the BCL-2/BAX ratio was not significantly reduced in Cis-treated cells when compared to untreated cells. Moreover, a minimal expression level of p-GSK3β/GSK3β was obtained in Cis alone, suggesting a low level of the inhibitory form of GSK3β. In a previous work, nuclear translocation of GSK3β can activate the NF-κB transcription factor in stimulating expression of anti-apoptotic (BCL-2) and antioxidant target enzymes (SOD and HO-1). This mechanism mediated by GSK3β resulted in photosensitizer Temoporfin resistance in HNSCC [10]. Additionally, accumulating evidence showed that GSK3β is relevant to chemoresistance in various cancers through regulating DNA repair, stemness of cancer cells, and NF-κB and AKT signals [25]. Hence, our findings support that GSK3β is involved in chemoresistance.

As previously reported, cell death induction caused by Cis depends on the generation of mitochondrial reactive oxygen species (mtROS), the presence of BAX and BAK, and caspase activation. Interestingly, when mtROS levels increased, resistant cancer cells were sensitized to Cis-induced cell death [26]. Similarly, AA inhibited cancer cell proliferation through ROS-mediated mitochondrial apoptosis and reduced GSH antioxidant levels in cancer cells [27]. This implies that antioxidant levels may affect apoptotic cell death and ROS production, as evidenced by the high susceptibility to oxidative stress observed in genetically *HO-1*-deficient mice [28]. Likewise, SOD-1 is a major antioxidant enzyme that catalyzes the conversion of superoxide radicals to hydrogen peroxide and oxygen. A previous study has shown that *Sod-1* deficiency induced intracellular superoxide radical accumulation in both the cytoplasm and mitochondria, leading to decreased proliferation and increased apoptotic cell death [29]. Based on these literature findings, the expression levels of *HO-1* and *SOD-1* were measured in SH-SY5Y cells treated with AA and Cis. As expected, the treatment with 20 µM AA in Cis-treated SH-SY5Y cells significantly downregulated the mRNA levels of *SOD-1* and *HO-1*, suggesting a potential anticancer mechanism through the downregulation of antioxidant gene expression.

AA alone and its combination treatment also exhibited dramatic effects in lowering the levels of *BCL2* gene expression and the BCL-2/BAX ratio, suggesting that AA or its combination could affect the regulatory proteins that led to apoptotic cell death of Cis-resistant SH-SY5Y cells. Furthermore, the combination had a significant effect, increasing the p-GSK3β/GSK3β ratio in the treated cells. It is likely that GSK3β inhibition was observed. A previous study demonstrated that inhibition of GSK3β was capable of inducing apoptosis through decreased expression of BCL-2 [30]. Therefore, apoptotic induction in the AA- and Cis-treated cells may be mediated by increasing GSK3β suppression. This possibility is supported by our observation that the combination treatment could downregulate *BCL-2, HO-1*, and *SOD-1* levels. Interestingly, this combined treatment also appeared to mediate GSK3β inhibition, suggesting that these two outcomes may be related. Although the exact mechanism by which GSK3β inhibition regulates *BCL2*, *HO-1*, and *SOD-1* gene expression remains unresolved in the present study, these findings lay the groundwork for future investigations.

Regarding molecular docking results, both GSK3β and BCL-2 are possible molecular targets of AA. The structure of GSK3β includes an N-terminal domain, a kinase domain that constitutes the ATP binding site and the enzymatically active site, and a C-terminal domain [31]. We have found that AA is bound to the protein kinase domain of GSK3β at residue PHE291 by hydrogen bonds. Additionally, it could also form a Van der Waals force with LYS205. In a previous work, deacetylation of LYS205 located in the kinase domain by SIRT1 contributes to the activation of GSK3β [32]. Therefore, considering the interaction, AA might be a potent GSK3β inhibitory agent in treating Cis-resistant cancer cells. Additionally, in a recent issue, 9-ING-41, a maleimide-derived GSK3β inhibitor, possessed anti-proliferative activity and the capacity to overcome chemoresistance [33] in different cancer cell lines. These are possible that GSK3β inhibitors might be applicable for cancers that are resistant to chemotherapeutic drugs. In addition to GSK3β, AA could interact with BCL-2 at residue ARG68, which is one of its potent active sites [34]. It is plausible that AA might be a potential inhibitor of BCL-2 in curing cancers with chemoresistance. Based on our observations, the RMSD values—used to evaluate the stability of the docked conformations compared to reference structures—were 0.624 Å and 0.061 Å for the interactions of AA with GSK3β or BCL-2, respectively. RMSD values below 2 Å indicate that the complexes are stable and reach equilibrium in their native states. Furthermore, the binding energies between AA and GSK3β or BCL-2 were −6.2 kcal/mol and −8.0 kcal/mol, respectively. These negative values reflect strong binding affinity, suggesting tight ligand binding to the target proteins and supporting the biological plausibility of these interactions. In parallel, Western blot findings showed that the AA and Cis treatment mediated inhibition of GSK3β. These experimental data support a plausible native interaction. Nevertheless, further comparison with additional experimental assays, such as direct binding assays, will be valuable.

**Fig 5:**
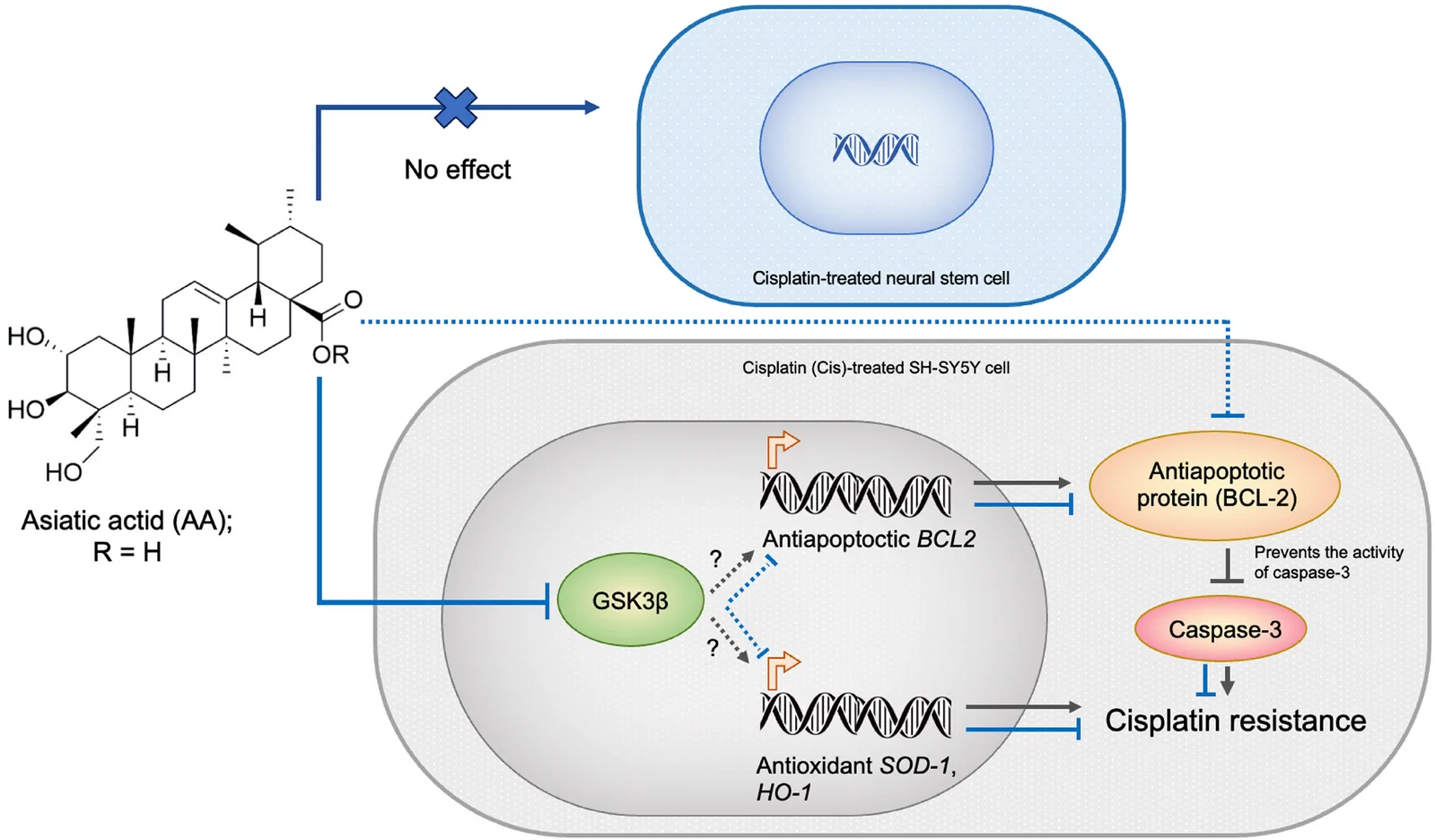
The schematic demonstrates the anticancer effects of AA on Cis-resistant SH-SY5Y and NSCs-hSCAPs. The grey lines indicate the potential Cis-resistant pathways in SH-SY5Y cells. The blue lines indicate the inhibitory effect of AA on Cis-resistant pathways in SH-SY5Y cells.

Considering the toxicity to non-malignant neural cells, AA did not alter the cell viability (%) of NSCs-hSCAPs treated with Cis when compared to the control. This suggested its low toxicity to the NSCs-hSCAPs and provided a selective effect on SH-SY5Y cells. In parallel, a previous work obviously revealed that AA could protect against the reduction of neurogenesis in the hippocampus, where neural stem cells are located, and memory deficits induced by valproic acid (VPA), and protect hippocampal neurogenesis impairments caused by 5-Fluorouracil (5-FU) chemotherapy [35]. Moreover, a previous work showed that inhibition of GSK3β in neurons had the ability to protect neuroprogenitor cells from genotoxicity and other stress-induced apoptosis [36,37]. This might help to explain how AA brought its safety effect to NSCs-hSCAPs. However, it needed further experiments to clarify. Taken together, the schematic diagram summarizes the findings shown in Fig 5.

## Conclusion

The combination of AA and Cis significantly decreased SH-SY5Y cell viability by enhancing apoptosis induction and reducing antioxidant gene expression. This was evidenced by the downregulation of *SOD-1, HO-1*, and *BCL2*, as well as the upregulation of *BAX* expression levels. Consistently, the treatment also led to a decreased BCL-2/BAX ratio, an increased percentage of apoptotic cells, and elevated caspase-3 activity. Furthermore, it exerted an inhibitory effect on GSK-3β. Thus, these outcomes may be functionally related. However, their mechanistic connections need further elucidation. Interestingly, *in silico* docking showed that AA plausibly interacted with multiple residues on both GSK-3β and BCL-2, suggesting its potential binding. Finally, AA did not affect the survival of Cis-treated NSCs-hSCAPs, indicating a favorable safety profile with low toxicity.

## Supporting information

S1 Raw Images Original images for blots. (PDF)

S1 Table CI index of Cis- and AA-treated NSCs-hSCAPs. (PDF)

S2 Table CI index of Cis- and AA-treated SH-SY5Y. (PDF)

S3 Table Band intensity for each protein obtained from SH-SY5Y. (PDF)

S4 Table Band intensity normalization with GAPDH. (PDF)

S5 Table Ratio data of BCL-2/BAX and p-GSK3β/GSK3β. (PDF)
