# Supplementary material for: Anticancer activity of asiatic acid in cisplatin-resistant human neuroblastoma SH-SY5Y and its unaffected effect on human neural stem cells
Source: PLoS One. 2026 Sep 15;21(9):e0358134. doi: 10.1371/journal.pone.0358134 (PMC13577367; doi:10.1371/journal.pone.0358134)
Supplement: Supplementary file 1 [file pone.0358134.s001.pdf]

S1_raw_images Original images for blots

Western blot results were obtained from three independent experiments. For each experiment, protein samples were resolved on two separate gels. The first gel was probed for BCL2 (26 kDa) or GSK3β (46 kDa), and the second was probed for BAX (20 kDa) and p-GSK3β (46 kDa). GAPDH (37 kDa) served as the internal control and was detected on both membranes. Chemiluminescent signals were captured using an Amersham™ ImageQuant 800 (Cytiva, MA, USA). Full, uncropped images of all original blots are provided.

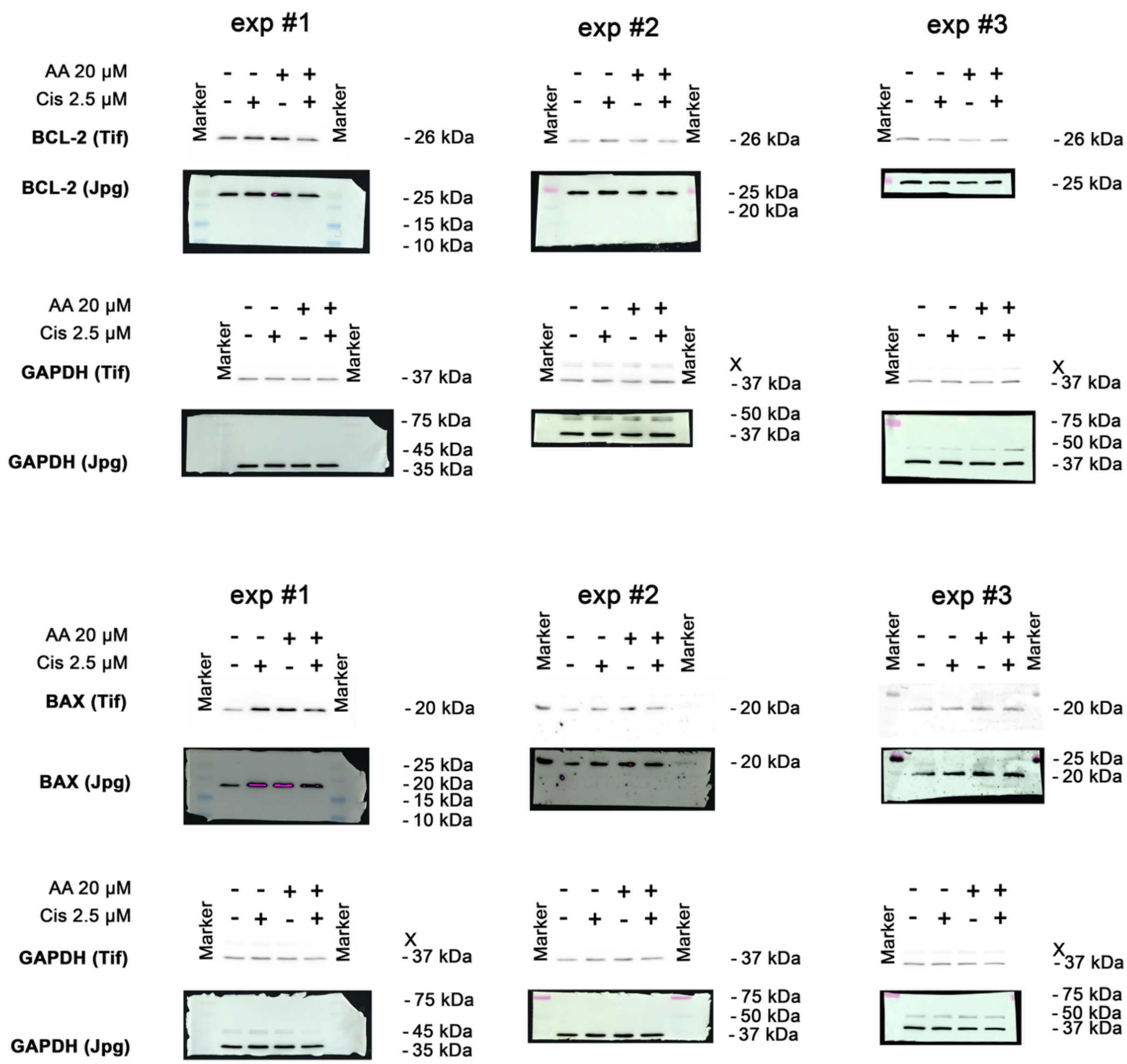

Original and uncropped Western blot images of BCL-2 (26 kDa), BAX (20 kDa), and GAPDH (37 kDa) in the tested groups, including control (AA 0 μM + Cis 0 μM), AA alone (AA 20 μM), Cis alone (Cis 2.5 μM), and combination (AA 20 μM + Cis 2.5 μM). X is a nonspecific band. Exp is experiment. The results were obtained from three independent experiments (exp #1, exp #2, and exp #3). Each membrane is expressed as TIFF and JPEG image formats. The TIFF files were preferred for scientific densitometry and quantitative analysis due to their uncompressed and lossless format.

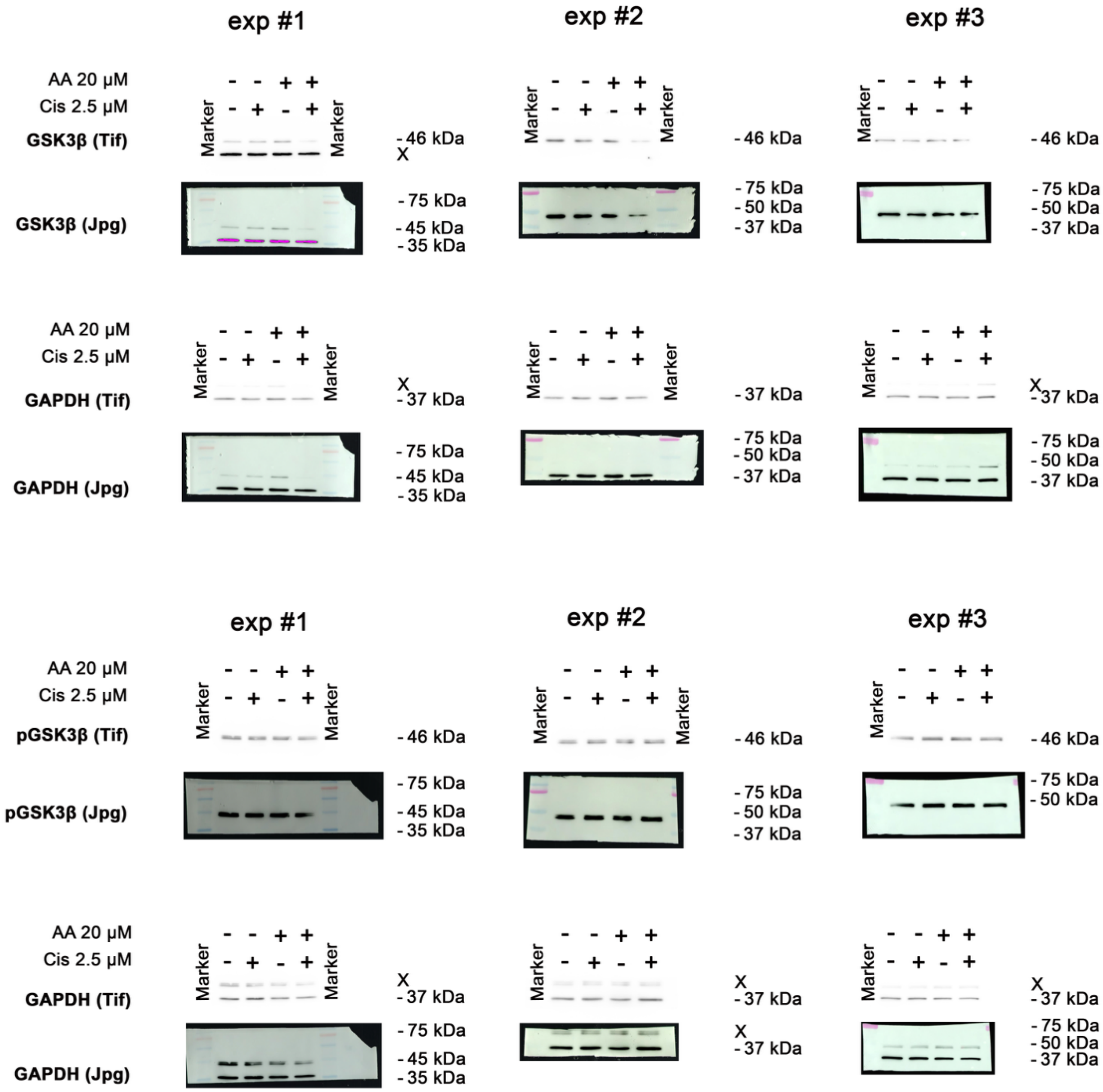

Original and uncropped Western blot images of GSK3β (46 kDa), p-GSK3β (Ser9) (46 kDa), and GAPDH (37 kDa) in the tested groups, including control (AA 0 μM + Cis 0 μM), AA alone (AA 20 μM), Cis alone (Cis 2.5 μM), and combination (AA 20 μM + Cis 2.5 μM). X is a nonspecific band. Exp is experiment. The results were obtained from three independent experiments (exp #1, exp #2, and exp #3). Each membrane is expressed as

TIFF and JPEG image formats. The TIFF files were preferred for scientific densitometry and quantitative analysis due to their uncompressed and lossless format.
