# Supplementary material for: Anticancer activity of asiatic acid in cisplatin-resistant human neuroblastoma SH-SY5Y and its unaffected effect on human neural stem cells
Source: PLoS One. 2026 Sep 15;21(9):e0358134. doi: 10.1371/journal.pone.0358134 (PMC13577367; doi:10.1371/journal.pone.0358134)
Supplement: Supplementary file 2 [file pone.0358134.s002.pdf]

S1 Table. CI index of Cis- and AA-treated NSCs-hSCAPs.

Cis (μM)	AA (μM)	Effect (%)	CI	Effects
20.0	1.0	25.53	1.18	antagonist
20.0	2.0	20.89	1.81	antagonist
20.0	10.0	20.20	1.95	antagonist
20.0	20.0	27.23	1.03	antagonist
40.0	1.0	33.39	1.27	antagonist
40.0	2.0	28.98	1.77	antagonist
40.0	10.0	31.20	1.49	antagonist
40.0	20.0	29.95	1.65	antagonist
80.0	1.0	48.99	0.87	synergistic
80.0	2.0	44.30	1.19	antagonist
80.0	10.0	50.64	0.78	synergistic
80.0	20.0	48.73	0.89	synergistic
