# Supplementary material for: Anticancer activity of asiatic acid in cisplatin-resistant human neuroblastoma SH-SY5Y and its unaffected effect on human neural stem cells
Source: PLoS One. 2026 Sep 15;21(9):e0358134. doi: 10.1371/journal.pone.0358134 (PMC13577367; doi:10.1371/journal.pone.0358134)
Supplement: Supplementary file 3 [file pone.0358134.s003.pdf]

S2 Table. CI index of Cis- and AA-treated SH-SY5Y.

Cis (μM)	AA (μM)	Effect (%)	CI	Effects
2.5	1	4.12	2.70	antagonist
2.5	2	10.38	0.93	synergistic
2.5	10	15.18	0.97	synergistic
2.5	20	36.76	0.41	synergistic
5	1	5.52	3.60	antagonist
5	2	14.17	1.12	antagonist
5	10	17.28	1.20	antagonist
5	20	37.67	0.50	synergistic
10	1	21.89	1.12	antagonist
10	2	23.22	1.05	additive
10	10	30.74	0.83	synergistic
10	20	54.39	0.33	synergistic
20	1	37.52	0.90	synergistic
20	2	35.71	1.00	additive
20	10	35.74	1.12	antagonist
20	20	38.23	1.13	antagonist
