# Supplementary material for: Anticancer activity of asiatic acid in cisplatin-resistant human neuroblastoma SH-SY5Y and its unaffected effect on human neural stem cells
Source: PLoS One. 2026 Sep 15;21(9):e0358134. doi: 10.1371/journal.pone.0358134 (PMC13577367; doi:10.1371/journal.pone.0358134)
Supplement: Supplementary file 4 [file pone.0358134.s004.pdf]

S3 Table. Band intensity for each protein obtained from SH-SY5Y.

Protein	Experiment No.	Band intensity			
		Control (Cis 0 μM + AA 0 μM)	Cis alone (Cis 2.5 μM)	AA alone (AA 20 μM)	Combination (Cis 2.5 μM + AA 20 μM)
BCL-2	1	9532.05	12402.24	11998.17	7994.39
	2	8710.78	12252.20	8036.90	7550.08
	3	9465.47	9465.47	9465.47	9465.47
GAPDH (for BCL2)	1	15841.19	16526.26	14023.07	14966.31
	2	9285.73	12931.22	14502.17	11308.56
	3	9146.20	9102.95	7497.64	6046.03
BAX	1	3680.88	12573.34	13620.63	9608.15
	2	3138.35	6411.25	10461.49	6288.02
	3	6520.73	9724.90	13133.70	11737.36
GAPDH (for BAX)	1	9413.73	11156.63	9976.63	8209.85
	2	7124.83	7559.02	7004.85	10327.02
	3	7554.47	7689.76	7166.93	8028.10
GSK-3β	1	5609.76	7476.95	10765.02	1757.79
	2	12761.46	8809.10	7683.66	1541.55
	3	9513.68	6121.37	8847.32	5304.64
GAPDH (for GSK-3β)	1	10263.90	8146.66	10058.20	7380.71
	2	9285.73	12931.22	14502.17	11308.56
	3	9146.20	9102.95	7497.64	6046.03
p-GSK-3β	1	20731.26	17928.43	18598.43	14347.00
	2	10827.00	13525.53	13373.72	14276.70
	3	9399.68	15960.75	14659.56	12540.15
GAPDH (for p-GSK-3β)	1	8490.78	9270.20	6069.83	5052.47
	2	7124.83	7559.02	7004.85	10327.02
	3	7554.47	7689.76	7166.93	8028.10
