# Supplementary material for: Anticancer activity of asiatic acid in cisplatin-resistant human neuroblastoma SH-SY5Y and its unaffected effect on human neural stem cells
Source: PLoS One. 2026 Sep 15;21(9):e0358134. doi: 10.1371/journal.pone.0358134 (PMC13577367; doi:10.1371/journal.pone.0358134)
Supplement: Supplementary file 5 [file pone.0358134.s005.pdf]

S4 Table. Band intensity normalization with GAPDH.

Protein	Experiment No.	Band intensity normalization with GAPDH			
		Control (Cis 0 μM + AA 0 μM)	Cis alone (Cis 2.5 μM)	AA alone (AA 20 μM)	Combination (Cis 2.5 μM + AA 20 μM)
BCL-2	1	1.00	1.25	1.45	0.89
	2	1.00	1.33	0.94	0.60
	3	1.00	0.67	0.39	0.58
BAX	1	1.00	2.89	3.50	3.00
	2	1.00	1.47	2.13	1.65
	3	1.00	1.50	2.46	2.72
GSK-3β	1	1.00	1.68	1.96	0.44
	2	1.00	0.50	0.39	0.10
	3	1.00	0.63	0.98	0.52
p-GSK-3β	1	1.00	0.79	1.26	1.16
	2	1.00	1.18	1.26	0.91
	3	1.00	1.70	1.90	2.01
