# Supplementary material for: Anticancer activity of asiatic acid in cisplatin-resistant human neuroblastoma SH-SY5Y and its unaffected effect on human neural stem cells
Source: PLoS One. 2026 Sep 15;21(9):e0358134. doi: 10.1371/journal.pone.0358134 (PMC13577367; doi:10.1371/journal.pone.0358134)
Supplement: Supplementary file 6 [file pone.0358134.s006.pdf]

S5 Table. Ratio data of BCL-2/BAX and p-GSK-3β/GSK-3β.

Protein	Experiment No.	Ratio data			
		Control (Cis 0 μM + AA 0 μM)	Cis alone (Cis 2.5 μM)	AA alone (AA 20 μM)	Combination (Cis 2.5 μM + AA 20 μM)
BCL-2/BAX	1	1.00	0.43	0.41	0.30
	2	1.00	0.91	0.44	0.36
	3	1.00	0.45	0.16	0.21
p-GSK-3β/GSK-3β	1	1.00	0.47	0.64	2.67
	2	1.00	2.37	3.25	9.14
	3	1.00	2.69	1.94	3.84
